# Malay translation and validation of modified checklist for autism in toddlers, revised with follow-up (M-CHAT-R/F)

**DOI:** 10.3389/fped.2024.1384292

**Published:** 2024-12-23

**Authors:** R. A. Ariffin, J. Ismail, F. N. Abd Rahman, W. S. Wan Ismail, N. Ahmad, A. Abdul Ghafar, W. W. Yang, F. Masra, N. Kamal Nor

**Affiliations:** ^1^ Child Development Centre (CDC), Department of Pediatrics, Universiti Kebangsaan Malaysia Medical Centre (UKMMC), Kuala Lumpur, Malaysia; ^2^ Department of Pediatrics, Faculty of Medicine, Universiti Sains Islam Malaysia (USIM), Nilai, Malaysia; ^3^ Department of Child and Adolescent Psychiatry Unit, Faculty of Medicine, UKMMC, Kuala Lumpur, Malaysia; ^4^ Department of Community Health, Faculty of Medicine, UKMMC, Kuala Lumpur, Malaysia

**Keywords:** conceptualization, data curation, formal analysis, funding acquisition, investigation, methodology, project administration, resources

## Abstract

**Introduction:**

Autism spectrum disorder (ASD) is a heterogeneous neurodevelopmental condition diagnosed clinically based on phenotypic characteristics and criteria such as the Diagnostic and Statistical Manual of Mental Disorders (DSM-5). Due to its significant social, emotional, and psychological impacts, early identification and diagnosis are crucial for starting early intervention and improving outcomes. A screening tool is imperative in identifying young children at risk so timely intervention can be instituted. The Modified Checklist for Autism in Toddlers, Revised with Follow-up (M-CHAT-R/F) is a reliable and valid screening tool used worldwide, with the previous iteration used for a long time in Malaysia. To enhance ASD screening in Malaysia, the latest version of M-CHAT-R/F was translated into Malay and evaluated for reliability and validity, as majority of the population speaks Malay, while the tool is originally in English. This study is a cross-sectional study performed in the Universiti Kebangsaan Malaysia (UKM) hospitals, between May 1st, 2020, to June 30th, 2022.

**Methodology:**

The English version of the M-CHAT-R/F was translated into Malay using forward and backward translation methods. Content and face validity were ascertained and a pilot study was performed for internal reliability. A total of 244 children attending clinics and wards in UKM hospitals aged 16-30 months were recruited based on three categories: children with typical development, suspected cases, and established cases of ASD. All caregivers of the recruited children were asked to complete the Malay M-CHAT-R/F. Reliability and validation assessments were performed.

**Results:**

Malay M-CHAT-R/F was found to be a reliable tool with good internal consistency (Cronbach's alpha = 0.906, *p* < 0.001). The Receiver Operating Characteristic (ROC) curve showed that cut-off scores of 2 on Malay M-CHAT-R/F lead to successful ASD classification with Area Under the Curve (AUC) = 0.887, *p* < 0.001 with a 95% CI (0.840, 0.933).

**Discussion:**

The assessment of the Malay M-CHAT-R/F showed satisfactory psychometric properties. Based on this study, the Malay M-CHAT-R/F is a reliable and valid screening tool to screen for ASD in children aged 16–30 months. Translating the M-CHAT-R/F into Malay is expected to improve community outreach and screening, which is essential for early diagnosis and timely intervention for children with ASD in Malaysia.

## 1 Introduction

Autism spectrum disorder (ASD) is a neurodevelopmental disability with a wide range of phenotypic heterogeneity which has been increasing in prevalence for the past few decades. Researchers from various disciplines have studied the preconception, pregnancy, and environmental causes of ASD, and although no single specific cause has been identified, various genetic, environmental, and gene-by-environment risk factors have been implicated (1). ASD is conceptualized by persistent impairments in social communication and interaction, and by restricted patterns of interest and behaviors (2, 3).

Worldwide, the prevalence of ASD has increased with greater parental awareness, community recognition, detection via medical screening programs, and improved accuracy of diagnosis by medical practitioners. A global prevalence study of autism reported a wide range of distribution across regions, with a median prevalence of 100/10,000. The World Health Organization has reported that ASD is detected in 1 out of 100 children. In the United States, the Centers for Disease Control and Prevention reported that autism currently affects 1 in 36 children aged 8 years (3, 4), representing a significant fourfold increase from 0.4% in 1996 to 1.68% in 2014 (5). In Malaysia, the number of children with autism registered with the Department of Social Welfare has increased by 7.6% over a decade, from 2013 to 2023, reaching a total of 53,323 registered children with ASD in 2023 (6). According to the National Autism Society of Malaysia (NASOM), there has been a 30% increase in cases seeking interventional program services with the organization across all age groups (7).

The diagnosis of ASD is made clinically and no biomarkers have been identified. Typically, a child is brought to be seen by a medical practitioner either due to a delay in development such as speech or socialization, or manifestations of specific behaviors. Diagnosis is made if the child fulfills criteria based on observation of specific behaviors, a history that delineates features of autism as well as the use of specific clinical, developmental, or psychological tools. Although ASD typically manifests within the first two to three years of life, children are sometimes not brought to be assessed by a medical practitioner until later in life, as sometimes parents and carers are not aware of ASD characteristics and what their implications are (4). Children and adolescents with ASD require substantial educational, vocational, and community support throughout their lives (5). Although parents may report initial concerns in children as young as 18 months or younger, most children in the United States receive a diagnosis only at 4 years and beyond (8) with non-English speaking countries reporting even older mean age of diagnosis. Early detection of ASD is crucial as it enables early intervention to be instituted. In neurodevelopmental conditions like ASD, there is a window of opportunity whereby intervention carried out during the optimal period is associated with much better outcomes due to brain neuroplasticity. Thus, late diagnosis may result in missing out on this window of opportunity to best impact the brain (9, 10).

Screening is key to preventative medicine. It distinguishes individuals who are at risk of the disease from those who are not. While screening tools are not diagnostic in nature, they facilitate the identification of at-risk individuals, allowing them to undergo diagnostic procedures earlier and at a less severe stage. Typically, ASD screening involves administering a parent-report questionnaire to prospectively identify ASD risk in children (11, 12). The challenges in diagnosing ASD are to detect children at risk of autism from the general population and to differentiate the overlapping symptoms of autism from other behavioral and developmental (OBD) disorders. This necessitates a fast, easy, cheap, and reliable screening tool for large population outreach. Globally, a multitude of screening tools exist yet none are recognized as a gold standard universally. The most widely used and studied is the Modified Checklist for Autism in Toddlers (M-CHAT), and its later version the Modified Checklist for Autism in Toddlers-Revised with Follow-up (M-CHAT-R/F) (13, 14). The M-CHAT-R has proven to be an efficient screening tool for toddlers, reducing the age of diagnosis by two years compared to the United States national median age of diagnosis (14, 15). With M-CHAT-R/F, it is possible to delineate children at high risk of ASD as early as 16 months of age and differentiate autistic characteristics from those who have typical development (TD) and OBD disorders (4, 16). Recognition, diagnosis, and appropriate intervention at younger ages are essential for better results in alleviating behavioral problems, building early life skills, reducing symptom severity, and achieving independence as the child grows (17).

As part of a growth and developmental surveillance initiative, Malaysia has been using the M-CHAT tool to screen for ASD. It was translated into Malay, the national language in Malaysia, and validated in 2019. It was included in the Baby and Children Health Record Book as part of the Malaysian national surveillance program and implemented as an ASD screening tool in toddlers between 18 and 30 months of age (16). The latest iteration of the M-CHAT, the M-CHAT-R/F, is an improvement as it can be used in children as young as 16 months, has fewer question items (from 23 in M- CHAT to 20 in M-CHAT-R/F), uses simpler language, and made clearer by providing examples after each question. M-CHAT-R/F also has better psychometric properties overall. M-CHAT-R/F is a two-step instrument; the M-CHAT-R is the parent-reported questionnaire with 20 “yes-or-no” questions and the Modified Checklist for Autism in Toddlers-Revised with follow-up (M-CHAT-R/F) includes second stage follow-up questions asked by doctors for children who fall into medium-risk category (failed 3 to 7 items) to obtain additional information from the failed M-CHAT-R items. Those who fall into mild (failed 0 to 2 items) or high-risk (failed 8 or more items) categories were not required to undergo the M-CHAT-R/F process (18). To improve ASD screening in Malaysia, the adoption of the M-CHAT-R/F and subsequent translation of the M-CHAT-R/F to Malay is necessary and a natural progression from previous screening strategies.

It is important to translate screening tools to the local language for nationwide use so that there is greater utilization of the tool, especially in suburban, rural areas and regions where English is not as widely used. To be user-friendly, the questionnaire must also be comprehensible and sufficiently straightforward for use by parents or caregivers for better demographic outreach. Part of translating a tool to the local language involves traditional forward-backward translation, which is translating a questionnaire from one language to another, and then translating it back to the original language to ensure accuracy. During the translational processes of M- CHAT-R/F to the Malay language, cultural adaptation was also considered by modifying several vocabularies and phrases to ensure they were culturally appropriate and meaningful for the Malay-speaking population while maintaining the integrity of the original tool. Translation with cultural adaptation involves not only linguistic translation but also consideration of cultural nuances, expressions, and sensitivities to ensure that the translated version is contextually appropriate and easily understandable in the target culture. This approach is expected to result in a more accurate and culturally sensitive adaptation of the M-CHAT-R, thus making it more effective in screening for ASD in diverse cultural contexts (9). It will be discussed further in the discussion category.

There are more than forty translated versions of M-CHAT-R/F. Countries such as Brazil, Mali, Indonesia, China, Taiwan, Albania, Serbia, and many more have translated and validated M-CHAT-R/F for use in their local population, with excellent sensitivity and specificity results. Medical practitioners’ use of M-CHAT-R/F is considered a level 1 screener, screening the entire population regardless of their ASD risk level. On the other hand, a level 2 screener such as Screening Test for Autism in Two-Year-Olds (STAT), is applied only by trained professionals to children who are already stratified as at-risk for whether they are more likely to have ASD rather than any other behavioral or developmental disorders (19).

This study aimed to translate M-CHAT-R/F into the Malay language and assess the reliability and validity of the Malay version of M-CHAT-R/F as a screening tool to diagnose ASD. Diagnostic tools such as the Diagnostic and Statistical Manual of Mental Disorders, Fifth Edition (DSM-5) for ASD and Childhood Autism Rating Scale, Second Edition Standard Form (CARS-2 ST) were used concomitantly to reach the final diagnosis after the screening questionnaire had been completed. Finally, it is hoped that with the Malay M-CHAT-R/F, the nationwide screening of ASD children in Malaysia could be updated and improved, and children with ASD in Malaysia can be detected earlier to enable them to undergo effective intervention and thus achieve better outcomes.

## 2 Materials and methodology

### 2.1 Study site and participants

This was a cross-sectional study performed at the Universiti Kebangsaan Malaysia (UKM) hospitals between May 1st, 2020 to June 30th, 2022 involving children aged 16–30 months. There were three categories of children: typical development children (TD), cases referred for developmental issues (R), and established ASD cases (E). The Child Development Centre (CDC), at Hospital Canselor Tuanku Muhriz, was initially used as the site for the study, and after the pediatric services moved to a new hospital, the CDC at the UKM Specialist Children's Hospital was used as the primary site. The ASD and R cases were recruited from the CDC, while the TD cases were recruited from UKM preschool/kindergarten, general pediatric clinics, and wards. The newly translated Malay M-CHAT-R/F was used as a screening tool to screen ASD risk and the results were stratified to low, intermediate, or high risk.

### 2.2 Ethical considerations

Research participation was voluntary, with written informed consent obtained before each recruitment. A small compensation for travel costs was given to research participants. All personal details were kept confidential. Diagnosis was revealed to parents and referrals were made for the child's hearing assessment and early intervention program including occupational therapy, speech therapy, and behavioral management program. Future appointments were given for follow-up and reassessment after interventions. This research was approved by the Ethics Committee of the Medical Faculty of UKM. Ethics code: TRANSLATIONAL-2019-001/1.

### 2.3 Translational process and pilot study for reliability and internal consistencies permission was obtained from the original author of the M-CHAT-R/F questionnaire for translation from English to Malay language

#### 2.3.1 Step 1: translation of the MCHAT-R/F from English into Malay language

For forward translation, three qualified translators including one language expert in both Malay and English languages, and two bilingual translators independently translated the M-CHAT- R/F from English to Malay after the original questionnaire was obtained. One Malay version of the M-CHAT-R/F was generated by each translator. The language expert gauged the choice of words used, phrased, formatted, and discerned any discrepancies in language as well as cultural context, and compared the two forward-translated Malay-language M-CHAT-R/F versions.

Two different bilingual independent translators who were not involved in the forward translation and who had not seen the original questionnaires were involved in the backward translation process from Malay to English. This backward-translated English language M- CHAT-R/F version and the original M-CHAT-R/F were compared.

#### 2.3.2 Step 2: content validity and comparability by expert review

The Malay translated version of M-CHAT-R/F was reviewed by two Developmental Pediatric Consultants from the CDC UKM for content validity and inspected for comparability with the original English M-CHAT-R/F ensuring accurate reflection from the original English content.

#### 2.3.3 Step 3: phase 1 pre-test (*n* = 5)

Phase 1 pre-test aimed to assess face validity which involved evaluating the comprehensibility of the content, language, question sequence, instructions, and layout as they were read. The translated Malay M-CHAT-R/F questionnaire was completed by five parents who were Malay native speakers with the ability to read and comprehend the Malay M-CHAT-R/F. The feedback received from all parents were gathered and any concerns with word misunderstandings and ambiguities were identified. The discrepancies in the translation were amended and enhanced.

#### 2.3.4 Step 4: amendments pre-final translated malay M-CHAT-R/F

The five participating parents who were involved in the face validity gave suggestions and ideas regarding their comprehension of the Malay M-CHAT-R/F. Vague wordings were modified and substituted. The comments provided by parents were utilized to establish both the face and content validity.

#### 2.3.5 Step 5: phase 2 pilot study (*n* = 30)

The translated Malay M-CHAT-R/F was administered alongside the original English version for pilot testing among 30 parents (20) who could read and comprehend both Malay and English languages. The participants were categorized into three primary cohorts: TD, R, and E. Once informed consent was obtained and demographic data gathered, the parents proceeded to complete all twenty questions in both the Malay and English versions of the M-CHAT-R/F. The confirmation of diagnosis for ASD was undertaken by developmental experts or child adolescent psychiatrists, utilizing the Diagnostic and Statistical Manual of Mental Disorders, Fifth Edition (DSM-5) for ASD and Childhood Autism Rating Scale, Second Edition Standard Form (CARS-2 ST). In instances where there was a disparity between the two diagnostic instruments, the final diagnosis was established by meeting the criteria outlined in the DSM-5 for ASD.

#### 2.3.6 Step 6: primary analysis

During this phase, the internal reliability of the translated Malay M-CHAT-R/F was assessed by comparing M-CHAT-R/F scores obtained from the Malay and English questionnaires. Analysis for the reliability of the data was performed using Cronbach's alpha.

**Figure 1:**
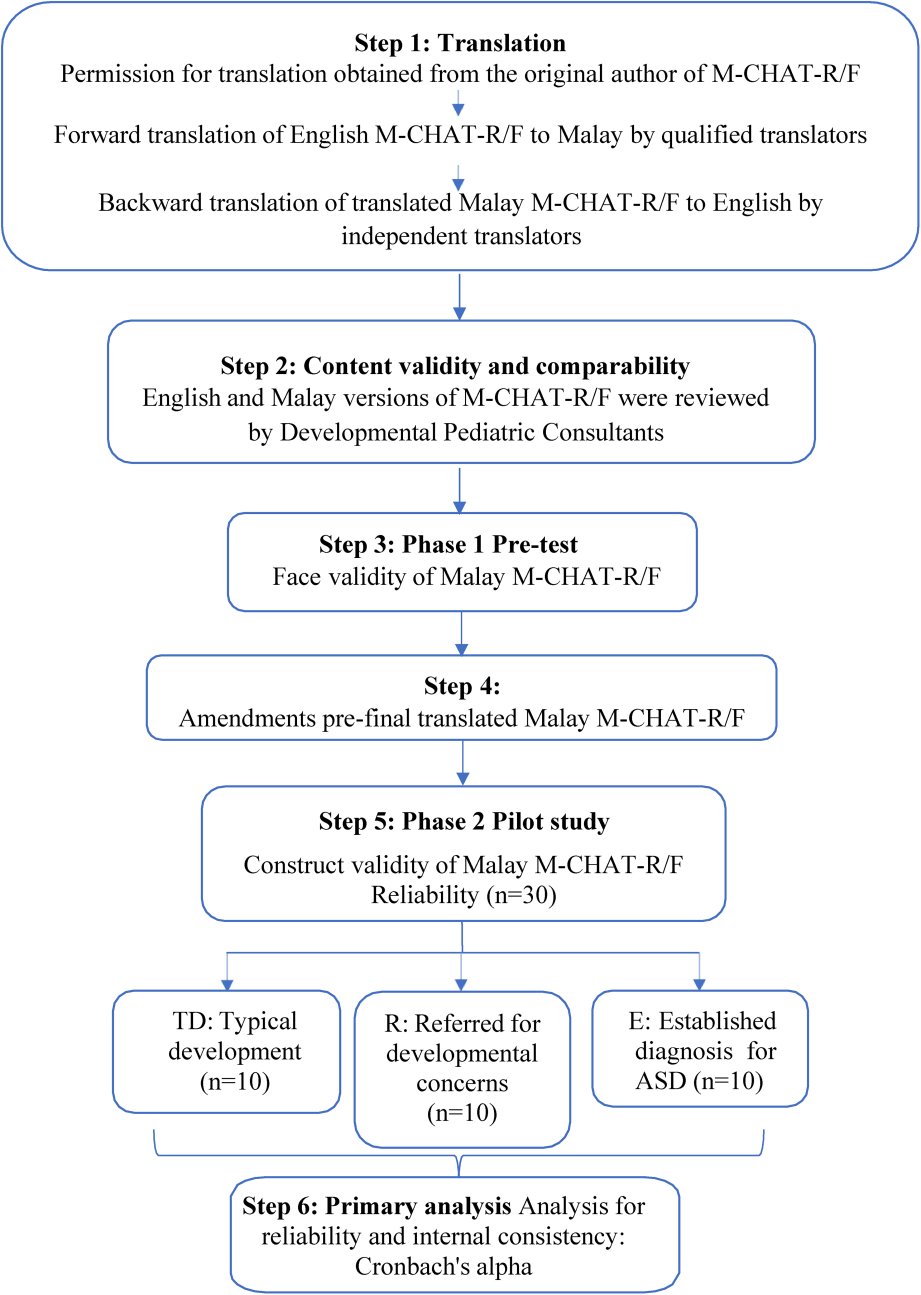
Translation and adaptation process of M-CHAT-R/F.

Figure 1 illustrates the translation and adaptation process of the M-CHAT-R/F.

### 2.4 Screening using the newly validated Malay version M-CHAT-R/F

#### 2.4.1 Step 1: participant recruitment

Children aged 16 to 30 months old were recruited using convenience sampling from the UKM preschool, pediatric wards, and pediatric outpatient clinics in UKM hospitals between the 1st of May 2020 to the 30th of June 2022. The participants were categorized into three groups as described in Step 5: Phase 2 pilot study. Once informed consent was obtained, the information sheet was provided and demographic data was gathered. The attending parent completed the Malay M-CHAT-R/F questionnaire independently while waiting in the pediatric clinics or wards.

This study's inclusion criteria encompassed children aged 16 to 30 months and their parents who could read and comprehend the Malay language. The exclusion criteria were children who were deaf or blind, children with severe motor disabilities such as cerebral palsy or neuromuscular diseases, and children with debilitating congenital, genetic, or metabolic illnesses.

#### 2.4.2 Step 2: sample size and data collection

The minimum sample size for sensitivity and specificity tests was calculated using Power Analysis and Sample Size (PASS) Software based on the assumption that all children with ASD would fail three or more out of the 20 items on the M-CHAT-R/F, as with the original scoring system. Including a margin of error of 20%, a total sample size of 240 (80 from each category TD, R, and E) was required to achieve a sensitivity of 90% and specificity of 95% with a *p*-value of <0.05 and power of 80%.

Based on the prevalence of the disease and both sensitivity and specificity of a screening diagnostic test in our outpatient clinic, a prevalence of 10% was used (21).

In this research, a total of 270 participants were initially recruited. Following the first recruitment phase, 20 participants were excluded. Subsequently, an additional 6 individuals were excluded during the eligibility evaluation, based on the predetermined inclusion and exclusion criteria. As a result, the final number of participants was finalized to 244 people.

### 2.5 Research tools

The questionnaire used in this study incorporated two main parts which were the (i) sociodemographic details of the patient and their parents and (ii) Malay-version M- CHAT-R/F questionnaire. In this research, only the Malay version of M-CHAT-R/F was used for validity. The researcher tabulated the results after the parent had completed all 20 questions. The average time taken to complete all questions was approximately 5 min. Parents were allowed to enquire with the researcher regarding any words or questions that needed clarification. Immediate collection and scoring of the questionnaire were conducted during the same setting.

All participants underwent diagnostic procedures with Developmental Pediatric Consultants, Child and Adolescent Psychiatrists, or trained General Pediatricians utilizing the DSM-5 for ASD and CARS-2 ST. Clinicians rated both tools based on information gathered from direct observation of the child and clinical histories obtained from parents. To use these tools, clinicians must have adequate exposure and training in ASD. DSM-5 for ASD consisted of two main components which were 1. social communication and interaction impairments (SCI) and 2. restricted, repetitive behaviors, interests, and activities (RRBs). Each participant must have persistent deficits in all three areas of component 1 (SCI) plus at least two of four areas of component 2 (RRBs). It also included specifiers of whether a child concomitantly has accompanying intellectual or language impairment, other neurodevelopmental, mental, or behavioral disorders, catatonia, or any association with known medical, genetic, or environmental factors (2). Following these assessments, all participants were diagnosed with ASD or no ASD. The severity of ASD was classified into three levels based on how much the impairments affected functioning: level 1 (minimal), 2 (moderate), or 3 (severe) (2). CARS-2 ST is a standard form used in younger children aged less than 6 years old or for those with lower-functioning ASD, compared to CARS-2 HF for those with high-functioning ASD. CARS-2 ST included fifteen items addressing multiple functionality abilities and each item was scored using a 4-point response scale. The total scores ranged from 15 to 29 for minimal to no symptoms of ASD, 30–36 for minimal to moderate symptoms of ASD, and >37 for severe symptoms of ASD (22, 23). ASD was diagnosed in children who scored 30 or higher. In the event of any differences, ASD diagnosis was ultimately determined by whether the child met DSM-5 criteria for ASD.

### 2.6 Data analysis

Analysis was performed using Statistical Package for Social Sciences (IBM SPSS Statistics v. 26). The results were presented as frequencies (n) and percentages (%) for categorical data, while the mean with standard deviation (SD) for continuous data. The chi-square test was used to analyze differences in categorical variables across groups comparing each variable from the demographic details. A *p-*value of <0.05 was considered statistically significant.

The translated Malay version was compared with the original for internal consistency using Cronbach's alpha.

The translated Malay version was assessed for psychometric properties; sensitivity and specificity, and positive and negative predictive values. The receiver operating characteristic (ROC) curve measured how well the Malay M-CHAT-R/F can detect ASD diagnosis in this study population. The area under the curve (AUC) varies between 0 and 1, where the perfect diagnostic accuracy is AUC = 1; very good AUC is between 0.9–1.0, good AUC is between 0.8–0.9, fair AUC is between 0.7–0.8, poor AUC is between 0.6–0.7, very poor AUC is between 0.5–0.6 and non- discriminating AUC is 0.5 (24).

In this study, the participants were first given the Malay M-CHAT-R/F questionnaire. Based on the final score, participants who fell into the moderate-risk of autism category i.e., total scores of 3 to 7, underwent a follow-up questionnaire for M-CHAT-R/F. Those who scored as low-risk of ASD (total score < 3) did not require any other action. Those who scored as high risk of ASD (total score > 7) did not require the follow-up questionnaire and went on to have CARS-2 and assessment for ASD including DSM-5 for ASD. We analyzed ROC for the Malay version of M- CHAT-R/F to assess any improvement in the area under the curve (AUC) as well as sensitivity, specificity, positive predictive value, and negative predictive values.

## 3 Results

### 3.1 Participants and characteristics

A total of 270 participants were recruited in this study. Of these, 20 participants were excluded due to various reasons: parents who did not proceed to the diagnostic procedure with DSM-5 and CARS-2 due to refusal or defaulting appointment. A total of 6 participants were excluded; due to gross motor disability (1 person), global developmental motor function delay (4 persons), and hearing impairment (1 person). There were 244 final study participants. Table 1 shows the demographic characteristics of the participants. There was no statistical difference in parental age nor average household income for both ASD and non-ASD categories. During the participants’ recruitment, the 53 children in the E group with established ASD diagnosis were already diagnosed with ASD before the screening procedure using Malay M-CHAT-R/F was performed. In both ASD and non-ASD groups, there were twice as many boys as girls, however, this was not statistically significant. Malay respondents outnumbered other racial groups with 89.8% made up of Malay participants while the rest were made up of Chinese (9%) and other ethnicities (1.2%) including East Malaysian locals (e.g., Bidayuh).

**Table 1:** Comparison of participant characteristics between children with and without ASD.

Characteristics	ASD (*n* = 90)	No ASD (*n* = 154)	*p-*value
Parent's details			
Parental age in years (mean, SD)			
Father	34.33 ± 5.81	35.03 ± 5.19	0.332^a^
Mother	32.80 ± 4.82	33.52 ± 4.91	0.267^a^
Household income in RM (*n*,%)	(mean = 6715.56, SD = 4115.74)	(mean = 6793.33, SD = 6140.66)	
0–4,849 (group B40)	33 (36.7)	59 (38.3)	0.226^b^
4,850–10,959 (group M40)	43 (47.8)	82 (53.3)	
10,960 and above (group T20)	14 (15.5)	13 (8.4)	
Children's details			
Sex (*n*,%)			
Male	65 (72.2)	98 (63.6)	0.169^b^
Female	25 (27.8)	56 (36.4)	
Race (*n*,%)			
Malay	84 (93.3)	135 (87.7)	0.347^b^
Chinese	5 (5.6)	17 (11.0)	
Others	1 (1.1)	2 (1.3)	
Age in months (*n*,%)	(mean = 19.65, S D = 2.34)	(mean = 27.46, SD = 1.95)	
16–23	13 (14.1)	72 (46.8)	<**0.001**^b^
24–30	76 (83.3)	83 (53.2)	
Siblings (*n*,%)			
0	2 (2.2)	2 (1.3)	0.824^b^
1	30 (33.3)	49 (31.8)	
≥2	58 (64.5)	103 (66.9)	
Past medical history (*n*,%)			
Present	17 (18.9)	23 (14.9)	0.804^b^
Absent	73 (81.1)	131 (85.1)	

^a^ Independent *t*-test.

^b^ Chi-square test. ***p* < 0.001** indicates statistically significant difference.

The participants were divided into two groups by age: younger group aged 16–23 months old and older group aged 24–30 months old. More than half of the research participants (64.3%) were between the ages of 24–30 months. There were more children with ASD in the older group (aged 24–30 months) compared to the non-ASD group (*p* < 0.001). Diagnosis of ASD was five-fold more prevalent in children of the older age group, whereas in those categorized as not having ASD, the distribution was almost equal between the two age groups. The mean age for ASD group was 26.8 (SD 4.5) months and for non-ASD group was 27.4 (SD 1.9) months. Parental age, household income, patient's sex and race, presence of siblings, or medical illnesses did not differ significantly between the two groups.

This study uses descriptive statistics with means, standard deviations, and frequencies to summarize the demographic data of the study population. These were chosen to provide an overview of the distribution of key variables. On the other hand, logistic regression was used to evaluate the association between the Malay M-CHAT-R/F results and ASD diagnosis with adjustment for potential confounders such as sex and participants’ age. In the logistic regression model, the children who failed the Malay M-CHAT-R/F were 3.5 times more likely to be diagnosed with ASD compared to those who passed the Malay M-CHAT-R/F (Odds Ratio (OR) = 3.5, 95% Confidence Interval (CI): 2.1–5.9, *p* < 0.001). Comparing the participants’ sex, male children had higher odds of being diagnosed with ASD compared to females (OR = 1.49, 95% CI: 0.84–2.63, *p* = 0.1692). In terms of age, younger children aged 16–23 months had significantly lower odds of being diagnosed with ASD compared to those aged 24–30 months (OR = 0.20, 95% CI: 0.10–0.39, *p* < 0.001).

Of the total participants recruited, 90 (36.9%) were typical development (TD), 101 (41.4%) were children referred for developmental problems (R) and 53 (21.7%) had established ASD diagnosis (E). The study population was delineated as those with ASD and those with no ASD. Medical practitioners diagnosed 90 (36.9%) of the children with ASD based on history, observation of behavior and interaction, and fulfilling DSM-5 criteria for ASD. There were 59 (24.2%) children who were identified as having OBD disorders as they had some behavioral or developmental difference but did not fulfill the diagnostic criteria using DSM-5, while 95 (38.9%) children were eventually categorized as TD. Of those in the OBD group, most were diagnosed as having isolated expressive speech delay and the rest with either global developmental delay, global developmental delay with autistic features, speech delay with autistic features, or social communication disorder with sensory issues (Table 2).

**Table 2:** Diagnosis categories for ASD and non-ASD.

ASD group (*n* = 90)	No ASD group (*n* = 154)	
Autism spectrum disorder (ASD), *n* = 90	Typical development (TD), *n* = 95	Other behavioral and developmental (OBD) disorders, *n* = 59: (a) Isolated expressive speech delay (*n* = 37) (b) Global developmental delay (GDD) (*n* = 2) (c) GDD with autistic features (*n* = 7) (d) Speech delay with autistic features (*n* = 11) (e) Social communication disorder with sensory issues (*n* = 2)

For the M-CHAT-R/F, a two-step screening was performed. Firstly, the Malay M-CHAT-R was filled by parents and secondly, M-CHAT-R/F was performed by the attending medical practitioner by clarifying with parents for those who were in the moderate risk category (scores 3–7). Table 3 shows the distribution of M-CHAT-R, M-CHAT-R/F, and CARS2-ST scores comparing children with and without ASD.

**Table 3:** Comparison of the screening procedures using malay M-CHAT-R, M-CHAT-R/F, and CARS2-ST scores between ASD and non-ASD groups.

Characteristics	ASD (*n* = 90)	No ASD (*n* = 154)	*p-*value
Malay M-CHAT-R score (*n*,%)			
Mild risk: 0–2	15 (16.7)	122 (79.2)	<**0**.**001**^a^
Moderate risk: 3–7	29 (32.2)	28 (18.2)	
High risk: 8–20	46 (51.1)	4 (2.6)	
M-CHAT-R/F score (*n*,%)			
Mild risk: 0–2	23 (25.6)	140 (90.9)	<**0**.**001**^a^
Moderate risk: 3–7	21 (23.3)	10 (6.5)	
High risk: 8–20	46 (51.1)	4 (2.6)	
Childhood Autism Rating Scale, 2nd edition, standard form (CARS2-ST) (*n*,%)			
Minimal: 15–29.5	11 (12.2)	150 (97.4)	<**0**.**001**^a^
Moderate: 30–36	39 (43.3)	4 (2.6)	
Severe: ≥37	40 (44.5)	0 (0.0)	

^a^ Chi-square test. ***p* < 0.001** indicates a statistically significant difference.

### 3.2 Reliability

The Malay M-CHAT-R exhibited excellent internal consistency with a value for Cronbach's alpha of 0.906 for internal consistency across all 20 items of the questionnaire between the original English M-CHAT-R/F with the Malay translated M-CHAT-R/F. This result exceeds the original instrument's reliability level (Cronbach's alpha of 0.79).

### 3.3 Psychometric properties of the M-CHAT-R/F: sensitivity, specificity, PPV, NPV

Of the 90 children diagnosed with ASD, 67 failed M-CHAT-R/F with a sensitivity of 74.4%; of the 154 children without ASD, 140 passed M-CHAT-R/F with a specificity of 90.9%. With regards to predictive values, of the 81 children that failed M-CHAT-R/F, 67 had ASD with a positive predictive value (PPV) of 82.7% and of the 163 children that passed M-CHAT-R/F, 140 had no ASD with a negative predictive value (NPV) of 85.9%. The psychometric properties of the Malay version of the M-CHAT-R/F are presented in Table 4.

**Table 4:** Psychometric properties of M-CHAT-R/F for the overall study population.

	Outcome	Diagnosis *n*, (%)		Total
		ASD	Not ASD	
M-CHAT-R/F	Failed	67 (74.4)	14 (9.1)	81
	Passed	23 (25.6)	140 (90.9)	163
Total		90	154	244

For the children with and without ASD, the M-CHAT-R/F scores were categorized by TD, R, and E categories as shown in Table 5. By using DSM-5 for ASD criteria, none of those from the TD group were diagnosed as ASD and none of those in the E group were not diagnosed as ASD. The M-CHAT-R/F was failed by 65.7% of those who already had an established ASD diagnosis (E), and 34.3% of referred cases (R). A large proportion of the referred cases (R), 78.6%, were not diagnosed as ASD but failed Malay M-CHAT-R/F. This shows that M-CHAT-R/F appears to detect developmental delay or behavioral issues and not only ASD. A large proportion of the referred cases (R) who were not diagnosed as ASD but failed Malay M-CHAT-R/F, mostly required reassessment as they either had moderate to high-risk Malay M-CHAT-R/F score or had a diagnosis of OBD disorders which warrant further follow-up and intervention.

**Table 5:** Comparison of M-CHAT-R/F results (pass vs. fail) in children with and without ASD (fail: score >2, pass score <3).

Diagnosis	ASD		Not ASD		
Malay M-CHAT-R/F Fail (score > 2) or Pass (score < 3)	Fail (*n*,%)	Pass (*n*,%)	Fail, (*n*,%)	Pass, (*n*,%)	Total
TD	0 (0)	0 (0)	3 (21.4)	87 (62.1)	90
R	23 (34.3)	14 (60.9)	11 (78.6)	53 (37.9)	101
E	44 (65.7)	9 (39.1)	0 (0)	0 (0)	53
Total	67	23	14	140	244

### 3.4 ROC curve

**Figure 2:**
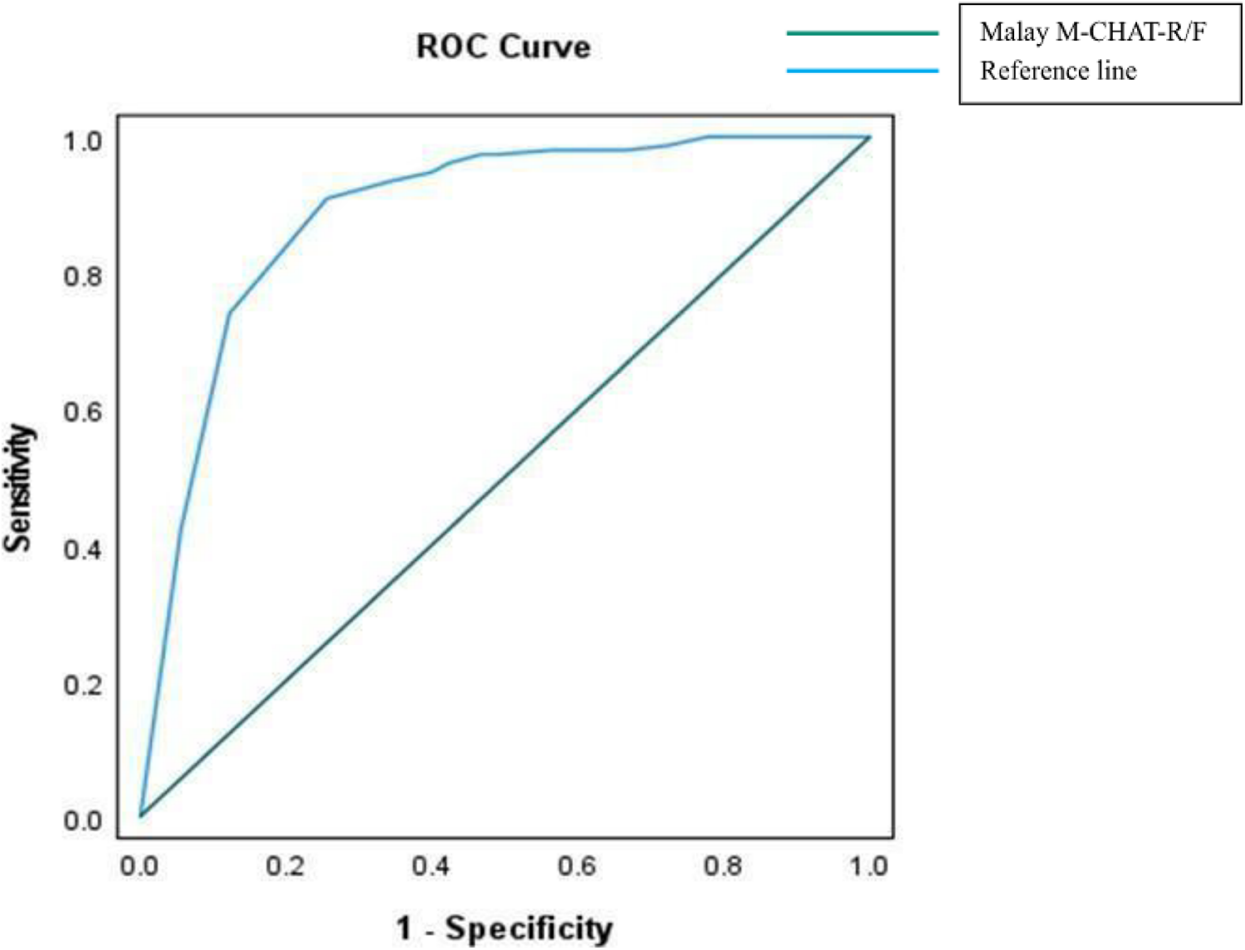
ROC curve for Malay M-CHAT-R/F.

Receiver operating characteristic (ROC) curve analysis suggests a successful classification of ASD based on the M-CHAT-R/F cut-off. Detecting ASD required a score that was as low as possible while minimizing missed cases. The area under the curve (AUC) represents the instrument's accuracy. The cut-off point score was at 2 items to reach a sensitivity of 66% and specificity of 90% for the Malay M-CHAT-R with area under the curve of 0.876 (*p* < 0.001). After applying second stage screening using M-CHAT-R/F, the outcome improved with a cut-off point at 2 items to reach a sensitivity of 74% and specificity of 88% with area under the curve calculated as 0.887 (*p* < 0.001) (Figure 2).

## 4 Discussion

This study was undertaken to assess the reliability and validity of the translated Malay M-CHAT-R/F for early screening of very young children. Our research study showed that the Malay M-CHAT-R/F was sensitive and highly specific when used as a screening tool to differentiate ASD and not ASD in Malaysian toddlers aged 16–30 months old, including in toddlers with typical development as well as in those with developmental and behavioral abnormalities. The Malay M-CHAT-R/F is a simple and quick tool to use, and in this study, parents could answer all 20 questions in less than 5 min. Another advantage of the M-CHAT-R/F is that it is a free item that can be easily downloaded and can be used by medical practitioners even without specific training. This study involved three different group categories: children with typical development with no parental concerns, those referred for developmental concerns, and those with a confirmed ASD diagnosis. The Malay M-CHAT-R/F screening was applied across these groups, but results should be interpreted within the context of this diverse sample.

The population screened in this research was recruited from a university hospital. Larger screening procedures for other areas in Malaysia, including those in rural areas where ASD detection is much lower, must be considered as screening in under-served areas suggests that positive rate differences could be attributed to socioeconomic, educational, and cultural factors (25, 26).

The psychometric properties of the Malay M-CHAT-R/F showed satisfactory results in screening a proportion of Malaysian children. This may not be generalizable to the whole nation but presents a promising starting point. The sensitivity of Malay M- CHAT-R/F was 74.4%; which was lower compared to the previous M-CHAT study by Lau et Al (16). with a sensitivity of 88.9%. We postulate that in contrast to Lau's study which did not include children with typical development, Lau's study specifically recruited children with ASD and OBD disorders, which likely resulted in higher sensitivity in that cohort. Ideally, in a standard screening validation study, participants should consist of a combination of children with typical development and children at various degrees of developmental risk, including those with established ASD to further enhance the tool's sensitivity. The inclusion of known children with established ASD in the cohort might result in potential sampling bias as they may not accurately reflect the general community that the screening tool is designed for. Nevertheless, the inclusion of this established ASD group enhances the sensitivity of the Malay M-CHAT- R/F questionnaire as it was proven to distinguish ASD from OBD disorders and TD children. In children who were referred due to neurodevelopmental concerns and did not fulfill the criteria of ASD, nearly 20% of the cases failed M-CHAT- R/F. Some of these children were found to have behavioral and developmental delays with or without autism features. As a screening tool, the Malay M-CHAT-R/F detects developmental problems not exclusively in children with autism. Comparing the Albanian and the original M-CHAT-R/F study by Robins et Al (18). reported a sensitivity of 85.4%; the lower sensitivity in this study is probably due to the smaller sample size and various sample characteristics (18, 26). To achieve higher sensitivity and maintain statistical power within each group, a larger sample size should be recruited in future studies, and a wider range of populations from various strata of society should be included. Furthermore, to improve the sensitivity estimates, these children with ASD risks or features and OBD disorders who were offered future evaluation in the CDC clinic should have a follow-up Malay M-CHAT-R/F re-evaluation and the data should be included in this study. This step also helps in reducing the rate of false negatives. Additionally, children with lesser symptoms were frequently detected at older ages (27).

On the other hand, the specificity of this study, at 90.9%, exceeds some of the other validity studies, for example, Lau et al. reported a specificity of 47.8% in 2019 (16), and a Brazilian M-CHAT-R/F study in 2021 reported specificity of 53.6% (24). A Chilean study in 2019 on the other hand quoted a specificity of 83.3% 2019 (28), while the original Robin et al. study reported a specificity of 99.3%. The high specificity of the Malay M-CHAT-R/F shows that those who pass the screening test are very unlikely to have ASD. In addition to being a more simplified version, Malay M-CHAT-R/F is likely to show far fewer positive results from developmental surveillance programs, which is important as this can reduce undue concerns and anxiety in parents, as well as alleviating the burden of unnecessary referrals to medical practitioners for further evaluations and diagnostic assessments.

Comparing various translations of the M-CHAT-R/F, there were large variations in PPV value across all the other translated M-CHAT-R/F studies. In this study, the PPV was 82.7%, whilst the M-CHAT-R/F studies in China reported a PPV of 91% (29), Taiwan 91% (30), Thailand 96% (31) while the original study reported PPV of 47% (18). In the Albanian study (2017), the internal reliability was 0.73 with PPV of 0.89 (26). Differences and discrepancies in results comparing translated M-CHAT-R and M- CHAT-R/F across different studies is most likely due to differences in methods and study populations such as using low-risk samples, a mix of low- and high-risk samples or only clinically referred high-risk samples (32). This may influence the classification of toddlers and the predictive validity indices. Our sample included children who had already been diagnosed with ASD and underwent screening and diagnostic assessments. This sampling bias is more likely present in tertiary clinical settings rather than community-based clinics. Additionally, parents of high-risk toddlers from referred or established cases of ASD may have answered differently from parents of children with typical development, either under-reporting the symptoms due to familiarity with the questions or the children may have improved after commencing intervention programs that may have ameliorated the autistic features or characteristics after diagnosis (33).

This study contributes to ASD research in the Southeast Asian population and considers local and cultural contexts. Some of the findings from this study are consistent with those reported in the literature from many other countries around the world, which consider the M-CHAT-R/F a useful screening tool for ASD. This translation and cultural modification of the MCHAT-R/F provides evidence-based ASD screening methods nationwide in Malaysia and possibly other regions that use the Malay language. The significance of cultural nuances should not be underestimated in the screening of ASD, as the condition is defined by recognizable patterns of behavior, social interactions, and communication, all of which are subjected to different cultural interpretations (34). Furthermore, identifying social communication difficulties often relies on deviations from culturally defined norms. For example, a perceived lack of eye contact may be interpreted as a significant symptom of ASD in Western cultures, where direct eye contact is expected, but it might be less relevant in some Asian cultures, where direct eye contact can be considered impolite (35).

The results of the study as a whole show that the Malay M-CHAT-R/F was sensitive and highly specific when used as a screening tool to detect the risk of ASD in Malaysian children aged 16–30 months old. Utilizing a reliable and valid screening tool in the local language is imperative as part of any nation's goal to detect conditions like ASD early, and to enable the identification and implementation of early intervention strategies to improve outcomes in children.

## 5 Limitations, applicability, and future directions

As proven by the recent study comparing forward-back translation and translation with cultural adaptation methods (9), more vigorous steps need to be incorporated to ensure comparability between the Malay and English M-CHAT-R/F. Equivalence testing by using statistical methods to test for equivalence across different language versions including confirmatory factor analysis (CFA) will increase the reliability of the translated M-CHAT-R/F questionnaire.

Standardized diagnostic instruments were not used to provide a sufficiently reliable assessment of ASD due to time constraints and limited resources. In the future, utilizing standardized tools like the Autism Diagnostic Observation Schedule (ADOS) may improve the reliability of the diagnosis.

The population screened in this research was recruited from one university hospital, thus the results may not be generalizable to the whole Malaysian population, and further refinements may be necessary. As the Malay M-CHAT-R/F is a screening tool, further research involving large, unselected community samples will be beneficial in the future to achieve accurate characterization of the instrument's validity and reliability for screening in Malaysian children in general. Large population targets may also be able to reach out to other racial groups and those from rural areas for better coverage and generalization of the Malaysian population. Due to the COVID-19 pandemic during part of the study, it could not be extended to other hospitals around Malaysia.

Additional data that may confer benefits in future studies would include details on the parent's socio-economic status, age at first parental concerns, and age at which the diagnosis was made. Furthermore, follow-up on children who need re-assessment of the M-CHAT-R/F especially those who portrayed autistic features and children with OBD disorders will provide more information on the result and final diagnosis. This will increase the sensitivity results in the Malay M-CHAT-R/F questionnaire.

## 6 Conclusion

ASD is a prevalent neurodevelopmental disorder both globally and in Malaysia. It has become more prevalent with earlier detection and population awareness. The heterogeneity has resulted in a conceptual expansion of the spectrum and traits of the disease. Regardless of the trajectory of these children's developmental milestones, the goal remains the same, which is to alleviate symptoms and get an accurate clinical diagnosis. Integrating the Malay M-CHAT-R/F into the developmental surveillance for children attending primary health care during well-child visits can facilitate early detection of ASD risk early, and potentially improve outcomes by enabling early intervention.

## Data Availability

The original contributions presented in the study are included in the article/Supplementary Material, further inquiries can be directed to the corresponding authors.
